# Estimating the association between being seropositive for cysticercosis and the prevalence of epilepsy and severe chronic headaches in 60 villages of rural Burkina Faso

**DOI:** 10.1371/journal.pntd.0007101

**Published:** 2019-01-24

**Authors:** Ida Sahlu, Hélène Carabin, Rasmané Ganaba, Pierre-Marie Preux, Assana Kone Cissé, Zekiba Tarnagda, Sarah Gabriël, Veronique Dermauw, Pierre Dorny, Cici Bauer, Athanase Millogo

**Affiliations:** 1 Department of Epidemiology, Brown University School of Public Health, Providence, Rhode Island, United States of America; 2 Department of Biostatistics and Epidemiology, College of Public Health, University of Oklahoma Health Sciences Center, Oklahoma City, Oklahoma, United States of America; 3 Department of Microbiology and Pathology, Faculty of Veterinary Medicine, University of Montréal, Saint-Hyacinthe, Québec, Canada; 4 Agence de Formation de Recherche et d'Expertise en Santé pour l’Afrique (AFRICSanté), Bobo Dioulasso, Burkina Faso; 5 INSERM, Univ. Limoges, CHU Limoges, UMR 1094, Tropical Neuroepidemiology, Institute of Neuroepidemiology and Tropical Neurology, Limoges, France; 6 Institute of Research in Health Sciences, Bobo Dioulasso, Burkina Faso; 7 Department of Veterinary Public Health and Food Safety, Faculty of Veterinary Medicine, Ghent University, Merelbeke, Belgium; 8 Unit of Veterinary Helminthology, Institute of Tropical Medicine Antwerp, Antwerp, Belgium; 9 Department of Virology, Parasitology and Immunology, Faculty of Veterinary Medicine, Ghent University, Merelbeke, Belgium; 10 Department of Biostatistics and Data Science, University of Texas Health Science Center at Houston, Texas, United States of America; 11 Centre Hospitalier Universitaire Souro SANOU, Bobo-Dioulasso, Burkina Faso; University of Texas Medical Branch, UNITED STATES

## Abstract

**Background:**

Individuals diagnosed with neurocysticercosis often present with epilepsy and sometimes with progressively worsening severe chronic headaches (WSCH). While cross-sectional associations between seropositivity to cysticercal antigens and epilepsy have been reported, few large scale studies have been conducted in West Africa and none have measured the association between seropositivity to cysticercal antigens and headaches. This study aimed at filling these knowledge gaps by estimating the strength of the cross-sectional association between seropositivity to cysticercal antigens and the prevalence of epilepsy and WSCH in 60 villages of Burkina Faso, West Africa.

**Methodology/Principal findings:**

Baseline data from a cluster randomized controlled trial collected from January 2011 to February 2012 in 60 villages across three provinces in Burkina Faso were used. Between 78 and 80 individuals were screened for epilepsy and WSCH in each village, and those screened positive were confirmed by a physician. Seventy-five percent of all participants were asked to provide a blood sample to test for *Taenia solium* cysticercus circulating antigens. Hierarchical multivariable logistic models were used to measure the association between seropositivity to cysticercal antigens and epilepsy (lifetime and active) as well as WSCH. Among 3696 individuals who provided a blood sample, 145 were found to have epilepsy only, 140 WSCH only and 19 both. There were positive associations between seropositivity to cysticercal antigens and active epilepsy (prevalence odds ratio (POR): 2.40 (95%CI: 1.15–5.00)) and WSCH (POR: 2.59 (1.34–4.99)).

**Conclusions/Significance:**

Our study is the first to demonstrate a cross-sectional association between seropositivity to cysticercal antigens and WSCH in a large community-based study conducted in West Africa. The measured cross-sectional association had a strength similar to the ones previously observed between seropositivity to cysticercal antigens and lifetime or active epilepsy. As a result, preventing new cysticercosis cases in communities may reduce the prevalence of these two important neurological disorders.

## Author summary

*Taenia solium* is a parasite that can be transmitted between humans and pigs in areas with poor sanitation and pig management practices. When this parasite infects the human brain, it can cause epilepsy or severe headaches. Our study aimed to measure the association between being seropositive to cysticercal antigens and having epilepsy (lifetime and active) or severe headaches in 60 villages of Burkina Faso. We found that active epilepsy and severe headaches were associated with seropositivity to cysticercal antigens. These results confirm prior studies which suggested an association between being seropositive for cysticercal antigens and these two neurological symptoms. Preventing cysticercosis could reduce new cases of these two neurological symptoms from occurring.

## Introduction

Epilepsy is reported to disproportionately affect rural low income countries [1], with lifetime epilepsy estimated to being nearly three times higher in rural developing countries compared to developed countries [1]. The prevalence of epilepsy is also reported to vary within sub-Saharan Africa (SSA) [2], potentially due to the distribution of risk factors across populations as well as variable impacts of selection and misclassification error biases in the published literature [2–4].

The prevalence of active headache disorders, in contrast to epilepsy, has consistently been reported to be the highest in high income countries [5]. In a review of 107 studies, the prevalence of current migraine was found to be highest in Europe at 15% and lowest in Africa at 5%, but the latter estimate was based on only seven studies [5]. Four community-based prevalence studies from SSA have reported one-year prevalence estimates for migraines and tension-type headaches ranging from 3.0% to 5.4% and 1.7% to 7.0%, respectively [6–9].

Neurocysticercosis (NCC) is suspected to play a role in the elevated prevalence of epilepsy in some low income countries [10]. NCC results from infection of the central nervous system with the larval stage of the tapeworm *Taenia solium* [11,12]. When humans or pigs harbor the larval stage of *T*. *solium*, the infection is referred to as human cysticercosis or porcine cysticercosis, respectively [13]. Human cysticercosis is acquired by ingesting *T*. *solium* eggs excreted in the feces of a human carrier of the adult cestode (i.e. taeniosis cases) [14]. Epilepsy and progressively worsening severe chronic headaches (WSCH) were shown in a meta-analysis to be the two most commonly reported neurological clinical manifestations among individuals with a diagnosis of NCC [15].

Two meta-analyses suggested that seropositivity to circulating antigens (current infection) or antibodies of cysticercosis (previous or current exposure to infection) were associated with epilepsy [16,17]. These meta-analyses demonstrated very limited evidence on the association between seropositivity to cysticercal antigens and epilepsy in community-based studies. Indeed, of the 38 unique studies included in these two meta-analyses, three used a combination of tests to detect seropositivity to cysticercal antigens and antibodies while only two focused on the association between seropositivity to cysticercal antigens and epilepsy [16,17]. Of those, three studies found a positive association, including the two studies that examined the association between seropositivity to cysticercal antigens only and epilepsy, but the small sample sizes resulted in wide confidence intervals [18–20]. Therefore, more studies with larger sample sizes evaluating the association between seropositivity to cysticercal antigens and epilepsy are needed.

The association between seropositivity to cysticercal antigens or antibodies, and headaches is even less studied. Only one community-based study conducted in Ecuador is available, which reported a prevalence odds ratio (POR) of 3.1 (95% CI: 1.1–8.5) between seropositivity to cysticercal antibodies and migraines [21]. A similar POR was found between the presence of intra-parenchymal NCC lesions and migraines [21]. No NCC lesions were found among those with tension-type headaches. In a more recent study, intraparenchymal calcified lesions of NCC were found to be associated with lifetime prevalence of headaches and intense headaches [22]. Hence, there is growing evidence of a possible association between cysticercosis and headaches, but no study has investigated the association between seropositivity to cysticercal antigens and headaches.

The aim of this paper is to examine whether the presence of *T*. *solium* cysticercus circulating antigens is associated with the prevalence of epilepsy and WSCH in a large community-based study conducted in 60 villages across three provinces in Burkina Faso. These results will help determine whether cysticercosis is a potential modifiable risk factor for epilepsy and WSCH.

## Methods

### Research design

This study is a prevalence case-control study of epilepsy and WSCH using the baseline cross-sectional component of a cluster randomized controlled trial aimed at estimating the effectiveness of an educational intervention to reduce the incidence of cysticercosis in humans [23]. The baseline participant recruitment and data collection took place from February 2011 to January 2012.

### Study sample

The aim of the sampling strategy was to screen 80 individuals for epilepsy and WSCH in each village, of whom 60 would provide a blood sample for the detection of circulating cysticercal antigens. One person per concession—a compound consisting of a chief and one to several households—was sampled, resulting in up to 80 individuals in 80 concessions sampled per village. The study took place in 60 villages across three provinces. Details on villages and concessions’ selection are provided elsewhere [24,25]. Briefly, 60 eligible villages located in 30 administrative departments were selected for future block randomization of the intervention [23].

### Selection of participants

In each selected concession, the chief was asked for consent and to enumerate all households, of which one was randomly selected. The selected household head was asked for his or her consent and to list all members older than 5 years of age living in the household along with information on which members had epilepsy. One household member was randomly selected and asked for consent. The first 60 consenting individuals were asked to provide a blood sample and to answer a screening questionnaire. If an individual did not consent to give a blood sample, he or she was invited to participate in the questionnaire component of the study. Once 60 individuals consented to provide blood samples, all other sampled individuals were invited to participate in the screening questionnaire only, until a total of up to 80 participants were enrolled in each village. All participants confirmed as having epilepsy or WSCH were also asked to provide a blood sample. Few refused to participate in the serological component of the study and all agreed to participate in the screening questionnaire component.

### Measurement and definition of epilepsy and severe chronic headaches

A screening questionnaire for epileptic seizures, epilepsy and WSCH was administered to all participants. The part of the questionnaire concerning epilepsy was based on the International League Against Epilepsy (ILAE) screening of epilepsy questionnaire developed by Preux *et al*. [26], and had been used by this research group in Burkina Faso in three villages in prior studies [18,27]. The questionnaire was further adapted to capture NCC-related headaches [28]. Individuals that screened positive were invited to be evaluated by a study physician, trained by a neurologist, for confirmation. In addition, 231 individuals screened negative were randomly selected to be examined by the physician in 37 villages. All physicians’ diagnoses were reviewed by the study neurologist. All confirmed cases of epilepsy and WSCH were invited to have a CT-scan, but results of this test will be reported elsewhere.

The primary outcomes of this study were neurologist-confirmed epilepsy and WSCH. Epilepsy was defined as more than one seizure of central nervous system origin without apparent cause in a lifetime [29]. Active epilepsy was defined as having had a seizure within the preceding five years, to allow for comparison across studies [19,30]. Individuals that did not meet the case definition of epilepsy were considered as epilepsy-free [31]. Six individuals diagnosed with single seizure only were excluded from all analyses. WSCH were defined as symptoms currently occurring more than once a week for more than two weeks, with each episode lasting for at least 3 hours and with a pain intensity that affected the ability to work, play, attend school or carry out usual activities or that requires analgesics [32]. Headaches also had to be progressively worsening in severity. This definition of WSCH was based on the headache definition by Jensen and Stovner [33] and adapted to capture characteristics specific to NCC [28].

### Measurement and definition of the primary exposure

The primary exposure was the presence of circulating cysticercal antigens in the blood as an indicator of the presence of viable cysticerci. The presence of circulating cysticercal antigens in the serum was measured using the B158/B60 enzyme-linked immunosorbent assay (Ag-ELISA) [34,35]. In a study conducted in Ecuador, this test was reported to have a sensitivity of 90% (95%BCI: 80–99%) and a specificity of 98% (95%BCI: 97–99%) to detect current cysticercosis [36]. In people with epilepsy, this test was reported to have sensitivities of 100% (95%CI: 54–100%) and 28% (95%CI: 13–47%) to detect active and inactive NCC lesions, respectively, while the specificity was estimated to be 83% (95% CI: 70–93%) for both[34]. Blood samples were collected by venipuncture and placed in 10 ml Venosafe serum gel tubes. Each sample was tested in duplicate wells of the ELISA. The mean Optical Density (OD) for each sample was obtained from the duplicate wells, which was then divided by a cut-off OD value to obtain a ratio. The cut-off OD was determined from a Student’s-t distribution with 7 degrees of freedom and a type I error of 0.001 using the mean and variance OD of eight negative control sera. The sample was considered seropositive for cysticercal antigens if the ratio was greater than 1 [37].

### Measurement of potential confounders and effect modifiers

The screening questionnaire gathered information on age, gender, ever attending school, occupation, pork consumption behavior, and history and knowledge of infection with the adult form of *T*. *solium*. The senior woman of the selected individual’s household was interviewed to gather information on pork preparation and consumption, and household members’ latrine use and access. The number of individuals identified by the chief of the household as having epilepsy was used as a proxy for measuring genetic epilepsy.

### Statistical methods

We first conducted descriptive analyses of the study population characteristics and of each outcome. In all models, individuals screening negative among those not seen by a physician and without confirmed epilepsy and WSCH among those seen by a physician were considered as not having the outcome of interest. We then estimated the crude associations between these characteristics and the prevalence of lifetime epilepsy, active epilepsy and WSCH using hierarchical logistic regression models with village-level and province-level random-effect intercepts. To account for the sampling design, all models adjusted for the type of concession sampled (see [24] for details).

To assess the relationship between seropositivity to cysticercal antigens and the two outcomes of interest while adjusting for confounding, we developed directed acyclic graphs (DAGs) with DAGitty [38] based on *a priori* knowledge. We considered the reported age, previous school attendance, gender, occupation, pork consumption, and latrine access reported by the mother as potential confounding variables. Using the DAGS, we determined the minimal set to adjust for confounding and measure the unbiased association between seropositivity to cysticercal antigens and both outcomes were: age, gender and whether the individual had ever attended school. In the model for epilepsy, we also adjusted for whether the individual came from a household with more than one individual with epilepsy. Age was categorized into three groups as 6–17 years, 18–40 years and 41 years and above.

Risk factors identified as associated with seropositivity to cysticercal antigens in Carabin *et al*., 2015 [25] were used to impute the values of missing Ag-ELISA results. The results of the models with and without imputed data for Ag-ELISA were similar, therefore we only present results without imputed data.

For the multivariable models, hierarchical logistic regression models were used to measure the association between seropositivity to cysticercal antigens and lifetime epilepsy, active epilepsy and WSCH adjusted for the confounding variables. We reported the POR and the associated confidence interval (95%CI).

All analyses were conducted with Stata 14.

### Ethical review

This study was approved by the Institutional Review Board at the University of Oklahoma Health Sciences Center and the ethical review panel at the Centre MURAZ (Burkina Faso). All participants were given written consent forms that were read by the field staff. Consent forms were signed or marked with an X or a fingerprint by those who were unable to write. All consent forms were signed by a witness. For those less than 16 years of age but 5 or greater, parents gave consent for their children. Assent was given by individuals aged 10 to 15 years. The field staff answered participants’ questions. As an incentive to participate in the study, all participants were given a bar of soap.

## Results

**Fig 1:**
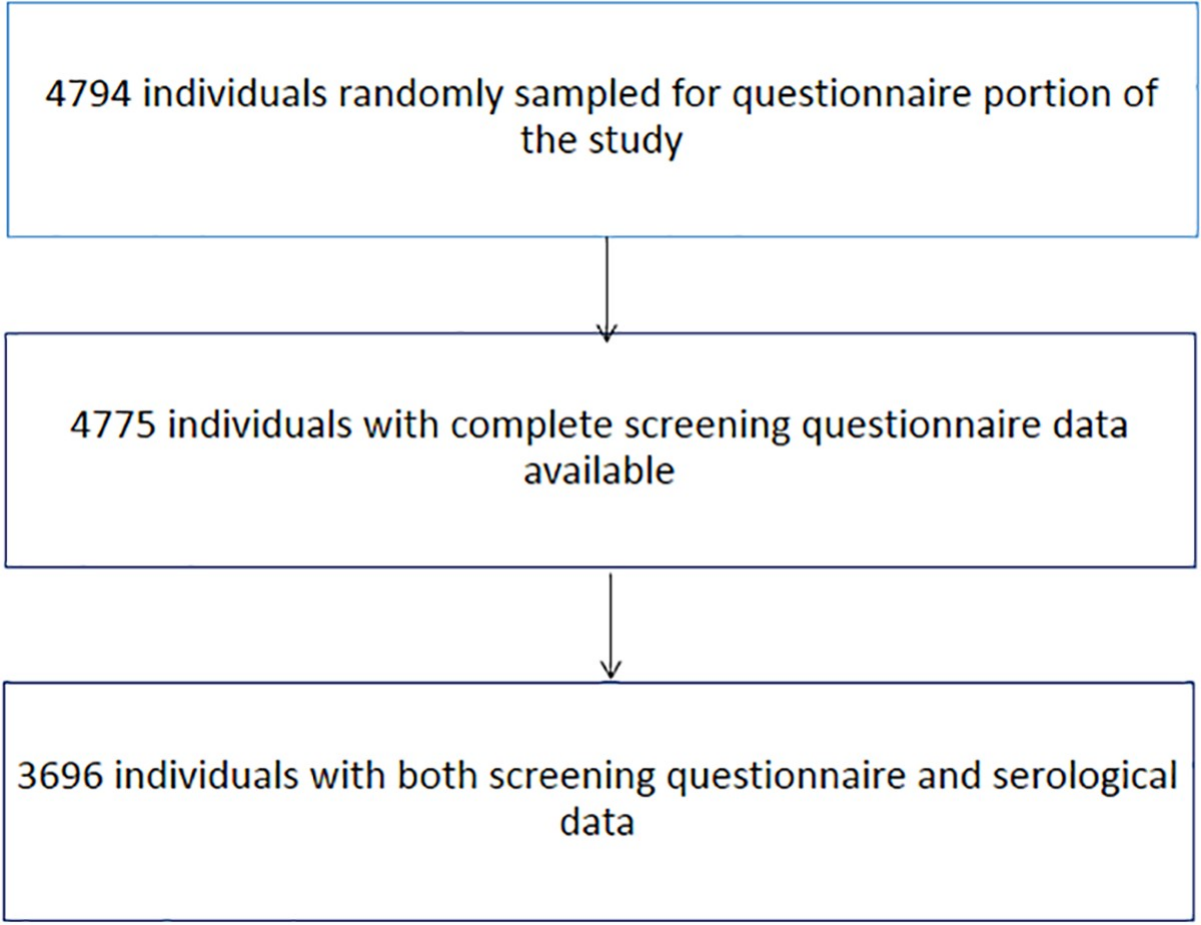
Flow chart of the study sample across 60 villages in three provinces of Burkina Faso, 2011–2012.

A total of 4794 participants consented to the questionnaire component of the study, 4775 answered all the screening questions among whom 3708 provided a blood sample for the detection of circulating cysticercal antigens. Six of the samples could not be analyzed due to small volume of serum available and an additional six with single seizures only were excluded, leaving 3696 individuals for the analysis (Fig 1).

A total of 614 individuals screened positive for epilepsy, WSCH or both, among whom 583 (95.0%) were examined by a medical doctor to confirm their diagnosis. Among those screened negative for epilepsy and WSCH, 201 were randomly selected for examination by a medical doctor to confirm their diagnosis.

Table 1 shows the characteristics of the study population. There were 145, 140 and 19 individuals diagnosed as having lifelong epilepsy, WSCH and both, respectively. These included 9, 16 and 2 individuals with confirmed lifetime epilepsy, WSCH and both, respectively, from individuals who had initially screened negative. Of the 164 people diagnosed with lifelong epilepsy, 125 (76.2%) had active epilepsy. The proportion of seropositivity to cysticercal antigens was 3.4% in the entire study population. There was a high proportion of individuals that had never attended school (70.4%) and did not have access to a latrine (87.9%). A small proportion of household chiefs reported that more than one person had epilepsy in their household (0.7%).

**Table 1:** Characteristics of 3696 individuals in the study population across 60 villages in rural Burkina Faso, February 2011-January 2012.

Variable	Characteristic	N (%)
**Confirmed neurological diagnosis**	Lifetime epilepsy	164 (4.4%)^a^
	Active epilepsy	125 (3.4%)
	WSCH^b^	159 (4.3%)
	None	3392 (91.8%)
**Seropositivity to cysticercal antigens**	Negative	3569 (96.6%)
	Positive	127 (3.4%)
**Province**	Boulkiemde	1834 (49.6%)
	Nayala	599 (16.2%)
	Sanguie	1263 (34.2%)
**Age (16 missing)**	6–17 years	1180 (32.1%)
	18–40 years	1281 (34.8%)
	41 years and older	1219 (33.1%)
**Gender (1 missing)**	Female	2033 (55.0%)
	Male	1662 (45.0%)
**Have you ever attended school? (1 missing)**	No	2602 (70.4%)
	Yes	1093 (29.6%)
**Occupation (1 missing)**	Student/Pupil	726 (19.7%)
	Farmer/gardener	1377 (37.3%)
	Housewife/house cleaner	1320 (35.7%)
	Commerce/ Salaried/ Skilled self-employed/ Unemployed	272 (7.4%)
**Access to a latrine (28 missing)**	No	3223 (87.9%)
	Yes	445 (12.1%)
**Where do you eat pork? (3 missing)**	Never ate pork	896 (24.3%)
	Eat pork at home only	1256 (34.0%)
	Eat pork in other home	429 (11.6%)
	Eat pork at the village market	596 (16.1%)
	Eat pork in another village market	215 (5.8%)
	Ate pork before, not now	301 (8.2%)
**Have more than one household member with epilepsy? (20 missing)**	No	3652 (99.3%)
	Yes	24 (0.7%)
**Type of concession**	Sow	500 (13.5%)
	Piglet	1395 (37.4%)
	Any type of concession	1801 (48.7%)

^a^ Includes the 125 individuals with active epilepsy and 19 individuals with both epilepsy and WSCH.

^b^ Includes the 19 individuals with both epilepsy and WSCH.

There were 11 (6.7%), 12 (7.6%) and 106 (3.1%) individuals who were seropositive to cysticercal antigens among those with confirmed lifetime epilepsy, WSCH and without confirmed epilepsy or WSCH, respectively. Table 2 shows the POR of lifetime and active epilepsy as well as of WSCH associated with seropositivity to cysticercal antigens from the univariable and multivariable hierarchical logistic models. In the univariable models, seropositivity to cysticercal antigens was associated with lifetime epilepsy (POR: 2.19 (95%CI: 1.14–4.19), active epilepsy (POR: 2.65 (95%CI: 1.34–5.23) and WSCH (POR: 2.68 (95%CI: 1.42–5.03)). In the multivariable models adjusted for the minimal set of confounders identified with the DAGs, the association between seropositivity to cysticercal antigens and active epilepsy (POR: 2.40 (95%CI: 1.15–5.00) and WSCH (POR: 2.59 (1.34–4.99)) decreased but remained statistically significant while the strength of the association for lifetime epilepsy became statistically non-significant (POR: 1.89 (95%CI: 0.94–3.79)). For lifetime and active epilepsy, adjustment for the minimal set has to be interpreted with care because, empirically, age, gender and school attendance were not associated with lifetime and active epilepsy among those seronegative to cysticercal antigens (i.e. the unexposed), making these variables non confounding variables in our dataset. A model only adjusting for the presence of more than one person with epilepsy in the household resulted in a POR of 2.18 (95%CI: 1.13–4.21) for lifetime epilepsy and 2.63 (95%CI: 1.32–5.26) for active epilepsy.

**Table 2:** Distribution of socio-demographic characteristics according to the neurological outcome and the associated prevalence odds ratios (POR) (95%CI) for lifetime epilepsy, active epilepsy and progressively worsening severe chronic headaches (WSCH) from univariable and multivariable hierarchical logistic models in 3696 individuals across 60 villages in rural Burkina Faso, February 2011-January 2012.

		No confirmed neurological outcome	Lifetime epilepsy			Active epilepsy			WSCH		
Characteristic		N (%)	N (%)	Crude POR (95%CI)	Adjusted POR^a^ (95%CI)	N (%)	Crude POR (95%CI)	Adjusted POR^a^ (95%CI)	N (%)	Crude POR (95%CI)	Adjusted POR^b^ (95%CI)
Seropositive to cysticercal antigens	Negative	3286 (96.9%)	153 (93.3%)	Reference	Reference	115 (92.0%)	Reference	Reference	147 (92.5%)	Reference	Reference
	Positive	106 (3.1%)	11 (6.7%)	2.19 (1.14; 4.19)	1.89 (0.94; 3.79)	10 (8.0%)	2.65 (1.34; 5.23)	2.40 (1.15; 5.00)	12 (7.6%)	2.68 (1.42; 5.03)	2.59 (1.34; 4.99)
Age (16 missing)	6–17 years	1120 (33.2%)	49 (30.3%)	Reference	Reference	37 (30.1%)	Reference	Reference	11 (7.0%)	Reference	Reference
	18–40 years	1172 (34.7%)	57 (35.2%)	1.15 (0.78; 1.71)	1.09 (0.72; 1.66)	49 (39.8%)	1.31 (0.84; 2.03)	1.20 (0.75; 1.92)	60 (38.0%)	5.24 (2.73–10.03)*	5.26 (2.67; 10.36)*
	41 years and older	1087 (32.2%)	56 (34.6%)	1.25 (0.84; 1.86)	1.04 (0.66; 1.64)	37 (30.1%)	1.06 (0.66; 1.69)	0.83 (0.49; 1.40)	87 (55.1%)	8.73 (4.62–16.52)*	10.08 (5.03; 20.17)*
Gender (1 missing)	Female	1845 (54.4%)	85 (51.8%)	Reference	Reference	65 (52.0%)	Reference	Reference	118 (74.2%)	Reference	Reference
	Male	1546 (45.6%)	79 (48.2%)	1.09 (0.80; 1.50)	1.11 (0.80; 1.54)	60 (48.0%)	1.09 (0.76; 1.56)	1.14 (0.78; 1.66)	41 (25.8%)	0.41 (0.29; 0.59)	0.39 (0.27; 0.57)*
Have you ever attended school (1 missing)	No	2366 (69.8%)	123 (75.0%)	Reference	Reference	93 (74.4%)	Reference	Reference	128 (80.5%)	Reference	Reference
	Yes	1025 (30.2%)	41 (25.0%)	0.77 (0.54; 1.11)	0.72 (0.47; 1.10)	32 (25.6%)	0.78 (0.52; 1.18)	0.71 (0.44; 1.13)	31 (19.5%)	0.55 (0.37–0.83)*	1.42 (0.90; 2.23)
Type of concession	Sow	455 (14.4%)	27 (16.5%)	Reference	Reference	20 (16.0%)	Reference	Reference	20 (12.6%)	Reference	Reference
	Piglet	1268 (37.4%)	72 (43.9%)	0.95 (0.60; 1.49)	0.92 (0.58; 1.46)	55 (44.0%)	0.98 (0.58; 1.66)	0.95 (0.56; 1.62)	61 (38.3%)	1.09 (0.65; 1.83)	1.04 (0.61; 1.76)
	Any	1669 (49.2%)	65 (39.6%)	0.66 (0.41; 1.05)	0.62 (0.39; 1.00)*	50 (40.0%)	0.69 (0.42; 1.17)	0.64 (0.38; 1.11)	78 (49.1%)	1.07 (0.64; 1.77)	1.03 (0.62; 1.73)
More than 1 household member with epilepsy (20 missing)	No	3359 (99.6%)	155 (94.5%)	Reference	Reference	118 (94.4%)	Reference	Reference	NA***	NA***	NA***
	Yes	14 (0.4%)	9 (5.5%)	12.99 (5.45; 30.97)*	15.02 (6.21; 36.36)*	7 (5.6%)	12.73 (5.00; 32.4)*	15.44 (6.01; 39.65)*	NA***	NA***	NA***
Occupation (1 missing)	Student/ pupil	698 (20.6%)	20 (12.2%)	Reference	NI**	16 (12.8%)	Reference	NI**	8 (5.0%)	Reference	NI**
	Farmer / gardener	1259 (37.1%)	72 (43.9%)	1.99 (1.20; 3.30)*	NI**	50 (40.0%)	1.75 (0.99; 3.10)	NI**	53 (33.3%)	3.57 (1.68; 7.57)*	NI**
	Housewife / house cleaner	1197 (35.3%)	46 (28.1%)	1.39 (0.81; 2.37)	NI**	37 (29.6%)	1.40 (0.77; 2.54)	NI**	86 (54.1%)	6.40 (3.07; 13.32)*	NI**
	Commerce / salaried / skilled self-employee	237 (7.0%)	26 (15.9%)	3.97 (2.16; 7.27)*	NI**	22 (17.6%)	4.15 (2.14; 8.07)*	NI**	12 (7.6%)	4.79 (1.92; 11.94)*	NI**
Access to a latrine (28 missing)	No	2958 (87.9%)	142 (87.1%)	Reference	NI**	107 (86.3%)	Reference	NI**	138 (86.8%)	Reference	NI**
	Yes	407 (12.1%)	21 (12.9%)	1.14 (0.71; 1.84)	NI**	17 (13.7%)	1.23 (0.72; 2.08)	NI**	21 (13.2%)	1.11 (0.67; 1.84)	NI**
Where pork consumption takes place (3 missing)	Never ate pork	820 (24.2%)	44 (26.8%)	Reference	NI**	34 (27.2%)	Reference	NI**	36 (22.6%)	Reference	NI**
	At home only	1191 (35.1%)	26 (15.9%)	0.36 (0.22; 0,60)*	NI**	20 (16.0%)	0.30 (0.16; 0.54)*	NI**	42 (26.4%)	0.77 (0.48; 1.24)	NI**
	In other home	399 (11.8%)	10 (6.1%)	0.38 (0.18; 0.77)*	NI**	5 (4.0%)	0.21 (0.08; 0.55)*	NI**	23 (14.5%)	1.17 (0.66; 2.07)	NI**
	At the village market	556 (16.4%)	19 (11.6%)	0.57 (0.32; 0.99)*	NI**	15 (12. 0%)	0.48 (0.25; 0.91)*	NI**	25 (15.7%)	0.97 (0.57; 1.67)	NI**
	At market of another village	191 (5.6%)	14 (8.5%)	1.13 (0.60; 2.14)	NI**	6 (4.8%)	0.51 (0.20; 1.27)	NI**	11 (6.9%)	1.24 (0.60; 2.54)	NI**
	Ate pork before, not anymore	232 (6.9%)	51 (31.1%)	3.58 (2.30; 5.57)*	NI**	45 (36.0%)	3.60 (2.19; 5.93)*	NI**	22 (13.8%)	2.15 (1.22; 3.78)*	NI**

^a^Adjusted for age groups, gender, school attendance, concession type and the number of people with epilepsy in the household as reported by the chief of the household.

^b^Adjusted for age groups, gender, school attendance and concession type.

* The 95% confidence interval does not include 1.

NI**: Not included

NA***: Not applicable

The strength of the associations between the socio-demographic variables explored and lifetime epilepsy, active epilepsy and WSCH are presented in Table 2. For the variables identified as being part of the minimal adjustment set in the DAGs, being an adult (18–40 years, and 41 years and above compared to 6–17 years), a woman and attending school showed statistically significant associations with WSCH but not lifetime or active epilepsy in the univariable hierarchical logistic models. Living in a household with more than one person with epilepsy was associated with higher prevalence odds of lifetime and active epilepsy compared to those who do not in the univariable models. In the multivariable models assessing the association between seropositivity to cysticercal antigens and lifetime and active epilepsy, only the effect of having more than one person with epilepsy was statistically significant. The effect of older age groups and being a female remained statistically significant in the multivariable model for WSCH, but school attendance became statistically non-significant, primarily due to the confounding effect of age and gender.

Of the other variables explored but not part of the minimal set in the multivariable models, individuals with an occupation other than being a pupil had a higher prevalence odds of WSCH and of lifetime epilepsy, but this was confounded by age. Declaring having eaten pork in the past but not anymore was associated with higher prevalence odds of both epilepsy and WSCH compared to those declaring never having eaten pork. Declaring eating pork at home only, in another home and at the village market was associated with higher prevalence odds of lifetime and active epilepsy, compared to those declaring never having eaten pork.

## Discussion

To our knowledge, this is the first study to examine the association between seropositivity to cysticercal antigens and two neurological disorders, epilepsy and WSCH, in a large community-based study in Sub-Saharan Africa.

Our study found a positive association between seropositivity to cysticercal antigens and epilepsy in the univariable hierarchical model. However, the strength of the association was weaker, albeit within the range, of that found in prevalence case-control studies conducted in three villages of Burkina Faso (POR = 3.4 (95%CI: 1.1–8.9)) and in one division of Burundi (POR = 3.8 (95%CI: 2.7–5.1)) [18,19]. Our estimate was however stronger than the association between the presence of antibodies to cysticercosis and epilepsy found in a prevalence case-control study conducted in Cameroon (OR: 1.3 ((95%CI: 0.6–3.0)) [19,39].

Unlike what was reported in two studies conducted in SSA [18,19], the strength of the association between seropositivity to cysticercal antigens and lifetime epilepsy became statistically non-significant in a model adjusting for the minimal set of confounding variables identified in the DAG. This decrease in the magnitude of the association might be partly due to our data since we did not observe a statistically significant association between gender, age and school attendance and the outcome among the non-exposed subjects, making this analysis possibly biased through over-adjustment [40]. However, the association remained significant for active epilepsy. The association between seropositivity to cysticercal antigens and active epilepsy, though previously reported, is somewhat counterintuitive since circulating antigens are typically detected when cysts are viable [13,34] while most NCC-associated seizures are believed to occur when cysts start degenerating [13]. However, NCC-associated seizures may also be the result of inflammation around cysts at the vesicular stage, especially in the case of massive infections [13]. Moreover, individuals with epilepsy may have had multiple cysts at different stages, either through re-infection or due to varying speeds at which cysts degenerate.

Our study is the first to report an association between seropositivity to cysticercal antigens and WSCH. Our prevalence estimate for WSCH was 4.3%. As previously discussed [23], since we aimed to capture NCC-associated WSCH, our definition of headaches was different from what had been used in other studies. However, our estimate was slightly higher, but within the range, to the lifetime migraine prevalence estimate of 3.3% (95%CI: 2.4% –4.6%) in Benin [41], and lower than the lifetime migraine prevalence estimate of 5.3% (95%CI: 5.0%– 5.6%) in Nigeria [42]. Only one prior study explored the association between seropositivity to cysticercosis antibodies, using enzyme-linked immunolectrotransfer blot testing, and migraines. In that cross-sectional study conducted in Ecuador, the POR was estimated at 3.1 (95%CI: 1.1–8.5) [21]. These results are consistent with our findings although [23] actually examining exposure to cysticercosis instead of seropositivity to cysticercal antigens as measured in our study.

The estimated POR remained very similar in our multivariable model adjusting for confounding variables identified with the DAG. In this case, however, age and gender were associated with the outcome among the unexposed, making these variables confounders in our data. Headaches might be caused by the presence of cysts in the ventricles or sub-arachnoid space which is associated with increased intra-cranial pressure or an inflammatory response [13]. Detection of cysticercal antigens in the cerebral spinal fluid or serum has been shown to be highly sensitive and specific for the detection of subarachnoid NCC [14] and active parenchymal lesions [34]. Our findings might be the result of cysts at different stages of development, either through re-infection or due to varying speeds at which cysts degenerate, or to extraparenchymal NCC. In addition, a recent matched prevalence case-control study conducted in one rural Ecuadorian village demonstrated a significant association between calcified NCC (without viable lesions) and lifetime, current and intense headaches [22]. This finding could suggest that WSCH are the result of infection with less cystic lesions or perhaps “older” active lesions. It is also be possible that circulating antigens remain in a few cases of calcified NCC, or that some antigens sporadically released by calcified cysts may be present in the serum of some cases with calcified NCC [43].

Our study has some limitations. First, the sampling design might have led to selection bias because concession sampling was based on the presence of pigs. We mitigated the effect of this bias on the PORs by adjusting for the type of concession in all of our models. Second, seropositivity to cysticercal antigens was used as a proxy for NCC. Serological tests for circulating cysticercal antigens are not very sensitive for the detection of non-viable parenchymal cysts and low number of cysts [44]. However, imaging such as CT-scans could only be offered to people with symptoms, and in the context of Burkina Faso, several people with symptoms refused to travel to receive imaging for fear of stigmatization. In our study, about 30% of individuals with confirmed epilepsy or WSCH did not get a CT-scan of the brain to measure NCC. Moreover, CT-scans are not very sensitive for the detection of live cysts or extraparenchymal cysts [45]. Therefore, seropositivity to cysticercal antigens allowed us to examine the association between seropositivity to cysticercal antigens and two neurological symptoms in the larger population. However, the associations observed for epilepsy and WSCH cannot be conclusively attributable to NCC. Third, we did not account for misclassification error of the exposure. We ran models that accounted for misclassification error for the outcome and did not find it to be an important bias in our study. Therefore, since our results were similar to other published studies, misclassification error of the exposure might also not be an important bias. Finally, the cross-sectional nature of our data did not allow for the assessment of temporality between the exposure and outcomes. Therefore, individuals with epilepsy and WSCH might have had their condition due to another cause that occurred prior to cysticercosis. In addition, it is possible that people with severe epilepsy are more at risk of acquiring cysticercosis due to increased exposure to contaminated food or the environment.

Our study also has several strengths. It is the first to report multivariable associations between seropositivity to cysticercal antigens for both epilepsy and WSCH. The few studies that examined the association between seropositivity to cysticercal antigens and epilepsy found a positive association, and we found that the association was very similar for active epilepsy. This study was also conducted in 60 villages and among more than three-thousand individuals, providing the power to detect associations between an exposure and outcome that were both relatively rare.

Our study supported the hypothesis that seropositivity to cysticercal antigens is associated with epilepsy and WSCH. Cohort studies examining sero-conversion to cysticercal antigens and its association with the development of these two neurological disorders are needed to better measure the magnitude of this association in rural communities of SSA.

## Supporting information

S1 Checklist STROBE checklist. (DOC)

S1 Dataset Table with individual level variables: Province, Village, school history, gender, age group, occupation, latrine access, pork consumption location, serological consent, household with more than one epilepsy member, concession type, screening result, physician examination, physician confirmation result, Ag-ELISA result, active epilepsy confirmation result. (XLS)
